# Targeting APOBEC3A to the viral nucleoprotein complex confers antiviral activity

**DOI:** 10.1186/1742-4690-4-61

**Published:** 2007-08-29

**Authors:** Ritu Goila-Gaur, Mohammad A Khan, Eri Miyagi, Sandra Kao, Klaus Strebel

**Affiliations:** 1 Laboratory of Molecular Microbiology, Viral Biochemistry Section, National Institute of Allergy and Infectious Diseases, NIH, Building 4, Room 310, 4 Center Drive, MSC 0460; Bethesda, MD 20892-0460, USA

## Abstract

**Background:**

APOBEC3 (A3) proteins constitute a family of cytidine deaminases that provide intracellular resistance to retrovirus replication and to transposition of endogenous retroelements. A3A has significant homology to the C-terminus of A3G but has only a single cytidine deaminase active site (CDA), unlike A3G, which has a second N-terminal CDA previously found to be important for Vif sensitivity and virus encapsidation. A3A is packaged into HIV-1 virions but, unlike A3G, does not have antiviral properties. Here, we investigated the reason for the lack of A3A antiviral activity.

**Results:**

Sequence alignment of A3G and A3A revealed significant homology of A3A to the C-terminal region of A3G. However, while A3G co-purified with detergent-resistant viral nucleoprotein complexes (NPC), virus-associated A3A was highly detergent-sensitive leading us to speculate that the ability to assemble into NPC may be a property conveyed by the A3G N-terminus. To test this model, we constructed an A3G-3A chimeric protein, in which the N-terminal half of A3G was fused to A3A. Interestingly, the A3G-3A chimera was packaged into HIV-1 particles and, unlike A3A, associated with the viral NPC. Furthermore, the A3G-3A chimera displayed strong antiviral activity against HIV-1 and was sensitive to inhibition by HIV-1 Vif.

**Conclusion:**

Our results suggest that the A3G N-terminal domain carries determinants important for targeting the protein to viral NPCs. Transfer of this domain to A3A results in A3A targeting to viral NPCs and confers antiviral activity.

## Background

APOBEC (*apo*lipoprotein *B *mRNA-*e*diting *c*atalytic polypeptide) proteins are a group of cytidine deaminases, which include APOBEC1 (A1), AID, APOBEC2 (A2), and a subgroup of APOBEC3 (A3) proteins in humans [1]. There are clusters of tandemly arrayed A3 genes present on human chromosome 22. These are A3A, A3B, A3C, A3DE, A3F, A3G, and A3H. In contrast, only a single A3 gene (mA3), which produces a protein with two Zn^2+^-binding motifs was found in mice [2]. Human A3G has been shown to be active against *vif*-defective human immunodeficiency virus type-1 (HIV-1) [3-13] and other viruses such as simian immunodeficiency virus, human hepatitis B virus, and HTLV1 [14-19]. In contrast, A3A was not found to inhibit HIV-1 but blocked replication of adeno-associated virus and retrotransposons such as intracisternal A particle (IAP) and long interspersed element 1 (LINE-1) [20-23].

A3G contains two copies of the cytidine deaminase active site (CDA) HXEX_23–28_PCX_2–4_C (where X is any amino acid) while A3A contains only a single CDA domain [1]. The cysteine and histidine residues are believed to coordinate a critical active site zinc ion while the glutamic acid residue participates directly in the deamination reaction [24]. Initial research suggested that this deamination activity was critical for APOBEC3-mediated inhibition of HIV-1 replication as A3G and A3F caused extensive mutagenesis of *vif*-defective HIV-1 proviruses [5-8,25-30]. More recent research has challenged this model based on the finding that some A3G and A3F mutants that appeared incapable of catalyzing deamination of deoxycytidine nevertheless retained substantial inhibitory activity against HIV-1 [31-34]. In addition, A3A mutants lacking the ability to induce cytidine deamination have been shown to effectively inhibit the mobility of retrotransposons [21-23].

In this study we wanted to investigate why A3A lacks antiviral activity against HIV-1. We observed that A3A was packaged into HIV-1 virions but did not associate with the viral nucleoprotein complex (NPC) and had no antiviral activity. In contrast, we previously reported that A3G, which exhibits strong antiviral activity, was packaged into viral NPC [35]. Sequence alignment of A3G and A3A revealed significant homology of A3A to the C-terminal region of A3G leading us to speculate that the inability to assemble into viral NPC may be due to the lack of an N-terminal CDA domain in A3A. To test this model, we constructed an A3G-3A chimeric protein, in which the N-terminal half of A3G was fused to A3A. This resulted in the creation of an enzyme containing two CDA domains. Interestingly, the A3G-3A chimera was packaged into HIV-1 particles and, unlike A3A, associated with the viral NPC. In support of our model, the A3G-3A chimera displayed strong antiviral activity against HIV-1 but was also sensitive to inhibition by HIV-1 Vif. These results suggest that the A3G N-terminal domain confers antiviral activity and Vif sensitivity to A3A and carries determinants required for the assembly into viral NPC.

## Results

### APOBEC3A has no antiviral activity and is insensitive to degradation by HIV-1 Vif

**Figure 1:**
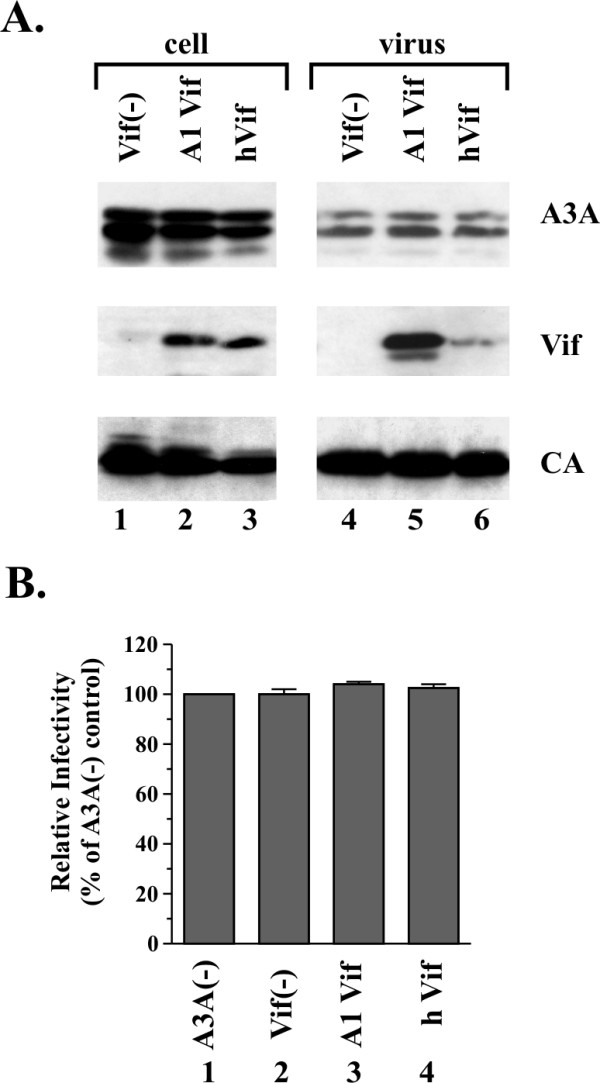
**A3A is resistant to Vif induced degradation**. **(A) **HeLa cells were transfected with vectors expressing *vif*-deficient pNL4-3 (3 μg each) along with pcDNA-A3A (1.5 μg each) and 1.5 μg of either pNL-A1vif(-) (lane 1), pNL-A1 (lane 2), or pcDNA-hVif (lane 3). Cells were harvested 24 h after transfection and whole-cell lysates were analyzed by immunoblotting using an A3G-specific rabbit polyclonal antibody (ApoC17) followed by incubation with an HRP-conjugated anti-rabbit antibody (A3A). The same blot was subsequently re-blotted with a Vif-specific monoclonal antibody (Vif) followed by probing with an HIV-positive patient serum to identify capsid protein (CA). Proteins are identified on the right. **(B) **Virus-containing supernatants from panel A were normalized for equivalent amounts of reverse transcriptase activity and used to infect LuSIV indicator cells [51] for determination of viral infectivity as described in Materials and Methods. Luciferase activity induced by virus produced in the absence of Vif and A3G was defined as 100% (lane 1). The infectivity of the remaining viruses was calculated relative to the control virus. Error bars reflect standard deviations from triplicate independent infections.

It has been reported that APOBEC3A (A3A) does not have antiviral activity towards HIV-1 irrespective of the presence or absence of Vif [20-22,25,27]. To verify these results, we tested the antiviral activity of human A3A and its sensitivity to HIV-1 Vif by transient transfection of HeLa cells. We used two different vectors for the expression of HIV-1 Vif: pNLA-1 Vif, expressing Vif together with other viral proteins from a proviral backbone [36], and pcDNA-hVif, expressing codon-optimized Vif [37]. Both forms of Vif can efficiently counteract the antiviral activity of A3G. HeLa cells were transfected with DNA encoding *vif*-defective HIV-1 and pcDNA-A3A together with either pNL-A1 (Fig. 1A, lanes 2 & 5) or pcDNA-hVif vector DNA (Fig. 1A, lanes 3 & 6) or empty vector (lanes 1 & 4). We found that neither expression of A1-Vif nor hVif reduced cellular A3A expression relative to the Vif-negative control (compare Fig. 1A, lanes 1–3). Furthermore, expression of Vif had no effect on the packaging of A3A into virus particles (Fig. 1A, compare lanes 4–6). We also compared the infectivity of viruses produced in the presence of A3A (Fig. 1B, lanes 2–4) to virus produced in the absence of A3A (Fig. 1B, lane 1) in a single cycle assay as described in Materials and Methods. Our data were consistent with previous reports and confirmed that A3A had no antiviral activity (Fig. 1B, compare lanes 1 & 2). Accordingly, the presence of Vif did not affect the infectivity of the viruses (Fig. 1B, lanes 3–4).

### Construction of APOBEC3G-3A chimera

**Figure 2:**
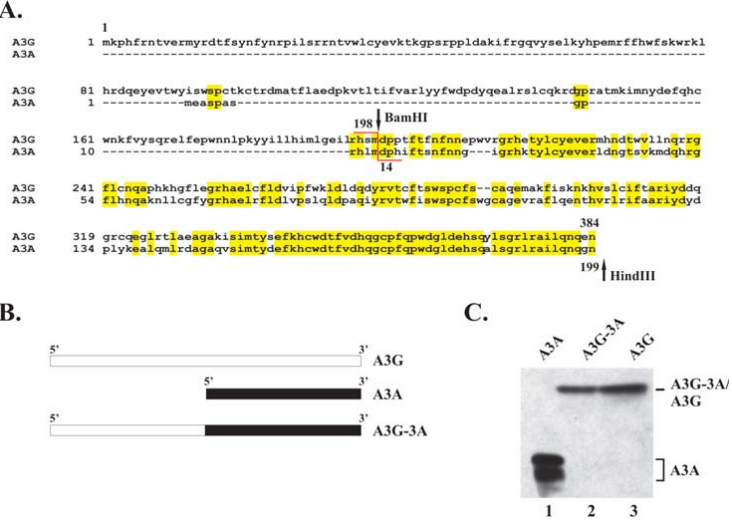
**Construction and expression of A3G-3A chimera**. **(A) **Sequence alignment of A3G and A3A. Highlighted areas indicate regions of amino acid identity. Arrows mark the location of unique BamHI and HindIII restriction sites in the expression vectors used for construction of the A3G-3A chimera. The chimera was constructed by replacing the BamHI and HindIII fragment in A3G by that of A3A. **(B) **Schematic illustration of the APOBEC expression vectors used in this study. **(C) **Expression of APOBEC proteins. HeLa cells were transfected with 5 μg each of pcDNA-A3A (lane 1), pcDNA-A3G-3A (lane 2), and pcDNA-A3G (lane 3). Total cell lysates were prepared 24 h after transfection and analyzed by immunoblotting for the expression of A3A, A3G-3A, and A3G, respectively using an A3G-specific polyclonal peptide antibody (ApoC17). Proteins are identified on the right.

It is well documented that APOBEC3G (A3G) has antiviral activity and is sensitive to inhibition by HIV-1 Vif [3-13]. Moreover, on comparing the amino acid sequences of A3G and A3A we found that A3A is highly homologous to the C-terminus of A3G (Fig. 2A). We therefore wanted to investigate whether the lack of A3A antiviral activity and the insensitivity of A3A to degradation by HIV-1 Vif were attributable to the lack of an N-terminal domain. We constructed an A3G-3A chimera by fusing the N-terminal domain of A3G to the N-terminus of A3A using a BamHI restriction site present in both A3G and A3A genes (Fig. 2A, BamHI). The resulting construct is schematically delineated in Fig. 2B. Expression of the A3G-3A chimera was analyzed by immunoblotting (Fig. 2C). For that purpose, HeLa cells were transfected with pcDNA-A3A (Fig. 2C, lane 1), pcDNA-A3G-3A (lane 2), or pcDNA-Apo3G (lane 3) and whole cells lysates were subjected to immunoblotting using an A3G-specific peptide antibody. Because of the high amino acid homology of A3A and A3G at their C-termini (Fig. 2A), the A3G antibody cross-reacted well with the A3A and A3G-3A proteins. A3A runs as a doublet on our gels. The reason for this is unclear but could be due to covalent post-translational modification of the protein or to initiation at an internal AUG codon.

### The APOBEC3G-3A chimera has antiviral activity

**Figure 3:**
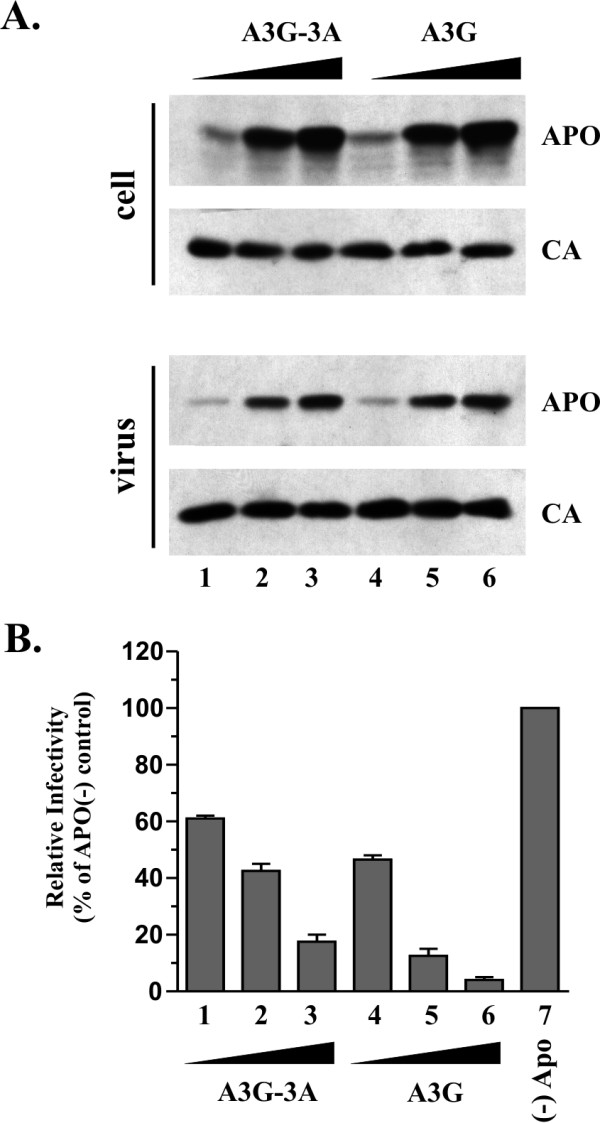
**The A3G-3A chimera has antiviral activity**. **(A) **HeLa cells were transfected with vectors expressing *vif*-deficient pNL4-3 (3 μg each) along with increasing amounts of pcDNA-A3G-3A DNA (lane 1, 1 μg; lane 2, 2 μg; lane 3, 3 μg) or pcDNA-A3G DNA (lane 4, 0.2 μg; lane 5, 0.5 μg, lane 6, 1 μg). Higher amounts of A3G-3A DNA relative to A3G DNA were chosen because A3G-3A was generally expressed at lower levels than A3G. The total amount of transfected DNA in each sample was adjusted to 6 μg using empty pcDNA3.1 vector DNA. Cells and virus-containing supernatants were collected 24 h post-transfection. Total cell lysate and concentrated virus preparations were analyzed by immunoblotting using an A3G-specific rabbit polyclonal antibody (ApoC17) followed by incubation with an HRP-conjugated anti-rabbit antibody (APO). The same blot was subsequently re-blotted with an HIV-positive patient serum (CA). **(B) **Virus-containing supernatants from panel A were normalized for equal reverse transcriptase activity and used to infect LuSIV indicator cells [51] for determination of viral infectivity as described in Materials and Methods. Luciferase activity induced by virus produced in HeLa cells in the absence of Vif and A3G was defined as 100% infectivity (lane 7). The infectivity of the remaining viruses was calculated relative to the control virus. Error bars reflect standard deviations from triplicate independent infections.

First, we wanted to test whether the A3G-3A chimera displayed antiviral activity against HIV-1. We transfected HeLa cells with *vif*-defective HIV-1 DNA along with increasing amounts of pcDNA-A3G-3A (Fig. 3, lanes 1–3) or pcDNA-Apo3G DNA (Fig. 3, lanes 4–6). Cell lysates (Fig. 3A, cell) and concentrated cell-free virus preparations (Fig. 3A, virus) were prepared 24 h after transfection and analyzed by immunoblotting using an A3G-specific antibody (Fig. 3A, APO). The same blot was then re-probed with an HIV-positive human patient serum (Fig. 3A, CA). As can be seen, A3G-3A and A3G exhibited similar mobilities in the gel, were expressed at similar levels, and were packaged into virus particles with similar efficiency and in a dose-dependent manner.

The infectivity of the viruses produced in figure 3A was analyzed in a single-cycle infectivity assay as described in Materials and Methods. Virus produced in the absence of A3G was included as a control and its infectivity was defined as 100% (Fig. 3B, lane 7). The infectivity of the other viruses was normalized for equal input virus and was expressed as percentage of the A3G-negative virus (Fig. 3B, lanes 1–6). As expected, packaging of A3G resulted in the dose-dependent inhibition of viral infectivity (Fig. 3B, lanes 4–6). Interestingly, the infectivity of viruses containing increasing amounts of the A3G-3A chimera was also reduced in a dose-dependent manner (Fig. 3B, lanes 1–3). These results demonstrate that, unlike A3A, the A3G-3A chimera has antiviral activity.

### HIV-1 Vif can reduce cellular expression and packaging of A3G-3A chimera

**Figure 4:**
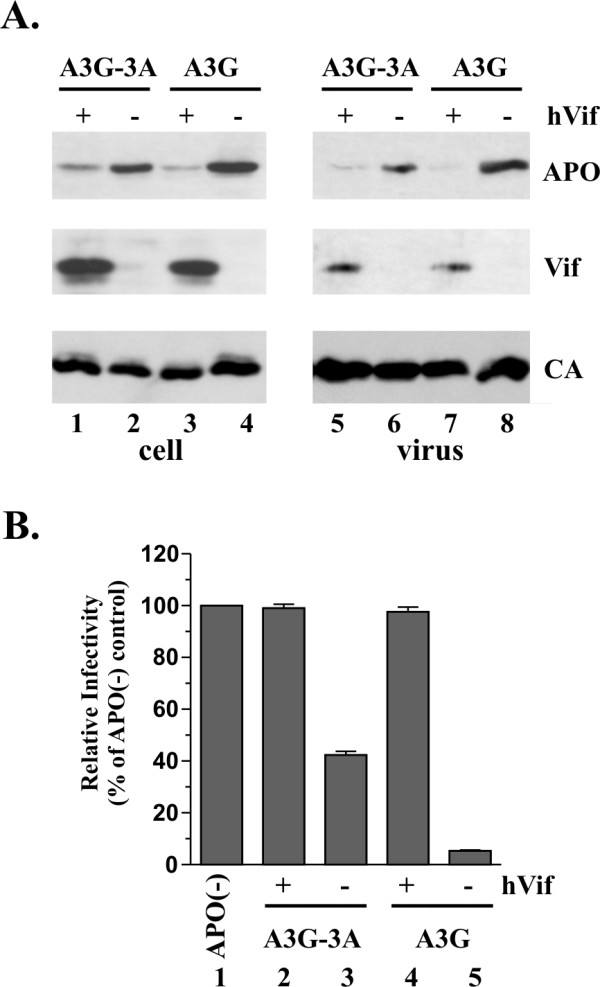
**A3G-3A is sensitive to HIV-1 Vif**. **(A) **HeLa cells were transfected with vectors expressing *vif*-deficient pNL4-3 (3 μg each) along with 1.5 μg each of pcDNA-A3G-3A (lanes 1–2, 5–6) or pcDNA-Apo3G (lanes 3–4. 7–8) as well as 1.5 μg pcDNA-hVif (+) or 1.5 μg empty pcDNA3.1 vector DNA (-). Cells and virus-containing supernatants were collected 24 h post-transfection. Total cell lysates and concentrated virus preparations were analyzed by immunoblotting using an A3G-specific rabbit polyclonal antibody (ApoC17) followed by incubation with an HRP-conjugated anti-rabbit antibody (APO). The same blot was subsequently re-probed with a Vif-specific monoclonal antibody (Vif) followed by an HIV-positive patient serum (CA). Proteins are identified on the right. **(B) **Virus-containing supernatants from panel A were normalized for equal reverse transcriptase activity and used to infect LuSIV indicator cells to [51] determine viral infectivity as described in Materials and Methods. Luciferase activity induced by virus produced in HeLa cells in the absence of Vif and A3G was defined as 100% infectivity (lane 1). The infectivity of the remaining viruses was calculated relative to the control virus. Error bars reflect standard deviations from triplicate independent infections.

HIV-1 Vif reduces cellular expression of A3G and inhibits packaging of A3G into virus particles. On the other hand, Vif neither affects the stability of A3A nor does it inhibit its encapsidation into HIV-1 virions (see Fig. 1A). We next investigated the sensitivity of A3G-3A to Vif-induced degradation and inhibition of virus-encapsidation. HeLa cells were transfected with *vif*-defective pNL4-3 DNA, along with pcDNA-A3G-3A (Fig. 4A, lanes 1–2 & 5–6) or pcDNA-Apo3G DNA (Fig. 4A, lanes 3–4 & 7–8) in the presence (odd lane numbers) or absence (even lane numbers) of pcDNA-hVif. Cell lysates and concentrated cell-free virus preparations were prepared 24 h after transfection and analyzed by immunoblotting using an A3G-specific antibody (Fig. 4A, APO). The same blot was then re-probed first with a monoclonal antibody to Vif (Fig. 4A, Vif) followed by an HIV-positive human serum (Fig. 4A, CA). We found that the A3G-3A chimera – like wt A3G – was sensitive to Vif-induced degradation (Fig. 4A, compare lanes 1–2 & 3–4). In addition, hVif inhibited the encapsidation of both wt A3G and the A3G-3A chimera (Fig. 4A, compare lanes 5–6 and 7–8). These results demonstrate that sensitivity to Vif is conferred to A3A by addition of the A3G N-terminal domain.

The infectivity of the viruses produced in figure 4A was analyzed in a single-cycle infectivity assay as described in Materials and Methods. The infectivity of virus produced in the absence of A3G and Vif (Fig. 4B, lane 1) was defined as 100% and used to calculate the relative infectivity of the remaining virus samples. Consistent with its effect on A3G and A3G-3A packaging, Vif efficiently inhibited the antiviral activities of A3G and A3G-3A (Fig. 4B compare lanes 1 to lanes 2 & 4). In contrast, the infectivity of viruses produced in the presence of A3G or A3G-3A but in the absence of Vif was significantly impaired (Fig. 4B, lanes 3 & 5). The less efficient inhibition of HIV-1 infectivity by A3G-3A when compared to A3G (Fig. 4B, lanes 3 versus 5) could be explained in part by the lower expression and encapsidation of A3G-3A relative to A3G in this experiment.

### The A3G N-terminal domain affects the subcellular distribution of A3A

**Figure 5:**
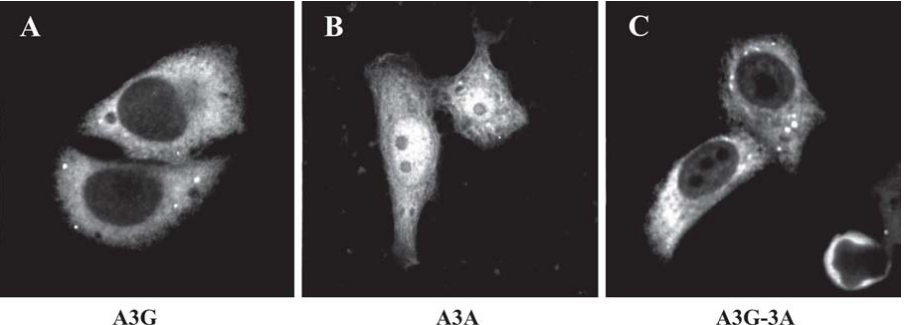
**Subcellular localization of APOBEC proteins**. HeLa cells were transfected with 5 μg each of pcDNA-Apo3G (panel A), pcDNA-A3A (panel B), or pcDNA-A3G-3A (panel C). Immediately following transfection, cells were detached from the flasks by trypsinization and re-seeded into 12 well plates containing microscope cover slips. Transfected cells were grown on the cover slips over night and then fixed with ice cold methanol for 10 minutes (-20°C). Cells were then stained with an A3G-specific rabbit polyclonal antibody (ApoC17) and analyzed by confocal microscopy as detailed in Materials and Methods.

A3G is largely localized to the cytoplasm where it can be found diffusely distributed or enriched in P bodies or stress granules [9,22,38-42] A3A, on the other hand, has been identified in both the nucleus and cytoplasm of transiently transfected cells [21-23]. To determine the effects of the A3G N-terminal domain on the cellular distribution of A3A, a side-by-side comparison of the intracellular localization of A3G, A3A, and A3G-3A was performed. HeLa cells were transfected with vectors encoding untagged A3G, A3A, and A3G-3A proteins. Immediately after transfection, cells were detached from the monolayer and re-seeded into 12 well plates containing microscope cover slips. Cell were grown on the cover slips for 24 h; then, cells were fixed with ice-cold methanol (-20°C, 10 min) and stained with A3G-specific peptide antiserum (Fig. 5). Consistent with previous studies, A3G exhibited predominantly cytoplasmic fluorescence (Fig. 5A). As predicted, A3A revealed nuclear and cytoplasmic staining (Fig. 5B). Interestingly, the subcellular distribution of the A3G-3A chimera largely reflected that of A3G (Fig. 5C). Thus, addition of the N-terminal domain of A3G to A3A induced a redistribution of the protein to a largely cytoplasmic localization. Punctate structures were observed in all samples and presumably represent P bodies or stress granules.

### The A3G-3A chimera associates with viral nucleoprotein complexes

**Figure 6:**
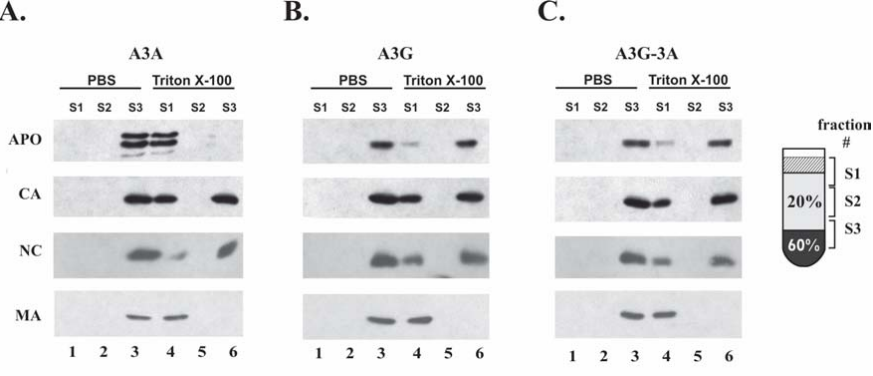
**A3G-3A co-purifies with viral nucleoprotein complexes**. Virus stocks were made in HeLa cells by cotransfection of pNL4-3 Vif (-) plasmid DNA (3 μg each) with 2 μg each of pcDNA-A3A (panel A), pcDNA-A3G (panel B), or pcDNA-A3G-3A DNA (panel C). Virus containing supernatants were collected 24 h post-transfection, filtered to remove cellular debris, and concentrated by pelleting through 20% sucrose. Viral pellets were suspended in 1 ml of DMEM and 500 μl each of the virus preparation was loaded onto a 20%/60% sucrose step gradient previously overlaid with 100 μl of PBS (lanes 1–3) or Triton X-100 (lanes 4 to 6) as described in Materials and Methods. Three fractions of 1.1 ml each were collected from the top of the gradient as shown in the cartoon on the right. Fraction S1 (lanes 1 & 4) contains soluble proteins; fraction S2 (lanes 2 & 5) is a buffer fraction of 20% sucrose that separates soluble proteins from virus particles or viral cores; fraction S3 (lanes 3 & 6) includes the interphase of 20%:60% sucrose where viral particles and viral cores accumulate. Gradient fractions were subjected to immunoblot analysis using an A3G-specific antibody (A3A, A3G, or A3G-3A) followed by probing with an HIV-positive patient serum (CA). Nucleocapsid protein (NC) was identified by a goat anti-NC antibody and matrix protein (MA) was identified by a mouse monoclonal antibody to MA(P17).

We have previously established that A3G is packaged into virus particles as a stable complex with viral NPCs [35]. We next wanted to test whether A3A and A3G-3A similarly assembled into viral NPCs. HeLa cells were transfected with DNA encoding *vif*-defective HIV-1 along with pcDNA-A3A (Fig. 6A), pcDNA-A3G (Fig. 6B), or pcDNA-A3G-3A DNA (Fig. 6C). Virus-containing supernatants were collected 48 h post transfection and concentrated by pelleting through 20% sucrose. Concentrated viruses were resuspended in 1 ml of DMEM and 50% each were loaded onto a 20–60% sucrose step gradient in the absence (Fig. 6, lanes 1–3) or presence of 0.1% Triton X-100 (Fig. 6, lanes 4–6). We have previously reported that components of the viral core, including nucleocapsid protein (NC), are resistant to 0.1% Triton X-100, whereas other viral components, such as matrix (MA) or envelope protein, are detergent sensitive and can be separated from core-associated proteins by sucrose step gradient centrifugation [43]. In this assay, intact viruses accumulate at the 20%/60% interphase of the step gradient column as evidenced by the enrichment of NC and CA protein in the S3 fraction (Fig. 6, lane 3). As expected, A3A, A3G, and A3G-3A partitioned with the viral fractions in fraction S3. No viral proteins were identified in fractions S1 and S2 attesting to the absence of soluble secreted proteins in our virus preparations. Detergent treatment resulted in the partitioning of CA and NC between the soluble S1 fraction and the detergent resistant viral core fraction S3 (Fig. 6, lanes 4 & 6). Interestingly, detergent treatment resulted in the quantitative sequestration of A3A to the soluble S1 fraction (Fig. 6A, lane 4) suggesting that A3A was not associated with viral NCPs. In contrast, >70% of the virus-associated A3G copurified with viral NPCs in fraction S3 (Fig. 6B, lane 6). Interestingly, A3G-3A behaved very similar to A3G and exhibited significant resistance to detergent extraction (Fig. 6C, lane 6). Thus, the A3G N-terminal domain imposed A3G-like properties onto A3A not only with respect to intracellular localization but also as far as packaging into viral NPC and antiviral properties were concerned.

## Discussion

It is well documented that human A3G has potent antiviral activity and effectively inhibits the replication of *vif*-deficient HIV-1. It is also accepted that the antiviral activity of A3G requires packaging of the protein into viral particles. Accordingly, wt HIV-1 is generally not susceptible to the antiviral properties of A3G since its Vif protein prevents A3G encapsidation. Recent reports demonstrated that A3A was able to inhibit LTR-retrotransposons and adeno-associated virus, a single-stranded DNA virus, but had no effect on *vif*-defective HIV-1 [20-23]. This was surprising since A3A, like A3G, was found to be packaged into HIV-1 particles and had deaminase activity [22]. Structurally, A3G and A3A differ by the presence of a second deaminase domain in A3G located in the N-terminal portion of the protein. Mutagenesis studies demonstrated a role of this N-terminal deaminase domain in A3G dimerization, Vif-sensitivity, and packaging into HIV-1 virions [44-48]. On the other hand, our data confirm that despite the lack of an N-terminal deaminase domain, A3A is efficiently packaged into HIV-1 virions (Figs. 1 &6). However, our data also show that encapsidation of A3A is qualitatively distinct from that of A3G: A3G is packaged into viral NPC and is resistant to detergent treatment while virus-associated A3A is detergent sensitive and does not co-purify with the NPC. This qualitative difference in packaging of A3G and A3A may well explain their different antiviral properties.

The reason for the lack of association of A3A with viral NPCs is unclear; however, we have previously shown that viral genomic RNA was required for the association of A3G with the viral NPC [35]. Importantly, A3G was still packaged into virus-like particles in the absence of genomic RNA; however, such A3G remained detergent sensitive [35]. Thus, we propose that functional packaging of APOBEC proteins into viral NPCs requires interaction with viral genomic RNA. Consistent with this model, A3A was packaged into virus particles irrespective of the presence or absence of viral genomic RNA (data not shown) suggesting that A3A lacks a domain required for the binding to viral genomic RNA. Thus, while A3A is packaged either non-specifically or via a specific interaction with viral component(s), it appears to lack a domain required for the specific assembly into viral NPCs. Interestingly, addition of the A3G N-terminal domain resulted in the targeting of the chimeric protein to viral NPCs. At the same time, the A3G-3A chimera acquired antiviral activity. These results suggest a correlation between the association of APOBEC proteins with the viral NPC and their ability to inhibit virus replication.

Consistent with the previously described importance of the A3G N-terminal domain for Vif-sensitivity, the A3G-3A chimera acquired sensitivity to degradation by Vif. Future experiments will investigate whether the regions in A3G determining Vif-sensitivity overlap with those required for NPC association. Also, A3A clearly differed from A3G and A3G-3A in its intracellular distribution (Fig. 5). The more prominent nuclear accumulation of A3A may explain its reported effects on retrotransposition. It will be interesting to define in more detail the regions in the A3G N-terminus affecting subcellular distribution of the A3A chimera. It is possible that the A3G N-terminus masks a nuclear import signal on A3A. Alternatively, the A3G N-terminus may contain a nuclear export signal preventing nuclear accumulation of the cytidine deaminase.

## Methods

### Plasmids

The *vif*-defective molecular clone pNL4-3Vif(-) [49] was used for the production of virus stocks. The construction of pcDNA-hVif for the expression of NL4-3 Vif from a codon-optimized gene under the transcriptional control of a CMV promoter has been described elsewhere [37]. Construction of pcDNA-Apo3Gmyc for the expression of C-terminally epitope-tagged wild type (wt) human A3G was reported elsewhere [4]. A variant, pcDNA-Apo3G, expressing untagged A3G was used for all of the experiments described in this study and was constructed by insertion of a stop codon at the end of the A3G gene in pcDNA-Apo3Gmyc [44]. pBluescript-APO3A was generously provided by Peder Madsen [50] and was used as template for PCR amplification of A3A using the 5' primer ATCAAGAATTCGGGACAAGCACATGGAAG and the 3' primer TTGTATAAGCTTCAGTTTCCCTGATTCTGGAG. The resulting PCR product was cloned between the Eco*RI *and Hind *III *sites of pcDNA3.1(-). pcDNA-A3G-3A was constructed by cloning a *Bam*HI and *Hind*III fragment from pcDNA-A3A into *Bam*HI and *Hind*III digested pcDNA-Apo3G. This strategy resulted in the in-frame fusion of A3G residues 1–197 to residues 14 to 199 of A3A (see Fig. 2A).

### Cell culture and transfections

HeLa cells were propagated in Dulbecco's modified Eagles medium (DMEM) containing 10% fetal bovine serum. LuSIV cells are derived from CEMx174 cells and contain a luciferase indicator gene under the control of the SIVmac239 LTR. These cells were obtained from Janice Clements through the NIH AIDS Research and Reference Reagent Program (Cat. no. 5460) and were maintained in complete RPMI 1640 medium supplemented with 10% FBS and hygromycin B (300 μg/ml). For transfection of HeLa cells, cells were grown in 25 cm^2 ^flasks to about 80% confluency. Cells were transfected using LipofectAMINE PLUS™ (Invitrogen Corp, Carlsbad CA) following the manufacturer's recommendations. A total of 5–6 μg of plasmid DNA per 25 cm^2 ^flask was generally used. Where appropriate, empty vector DNA (pcDNA3.1(-)MycHis (Invitrogen)) or *vif*-defective vector DNA (pNL-A1vif(-)) was used to adjust total DNA amounts. Cells were harvested 24 h post-transfection.

### Antibodies

A peptide antibody to human A3G was prepared by immunizing rabbits with KLH-coupled peptides corresponding to residues 367 to 384 of human A3G. A goat anti-NC(p7) polyclonal antibody was a gift of Robert Gorelick. Viral matrix (MA) protein was identified by a mouse monoclonal anti-MA(p17) antibody (Cellular Products Inc. Buffalo NY). A monoclonal antibody to Vif (MAb #319) was used for all immunoblot analyses and was obtained from Michael Malim through the NIH AIDS Research and Reference Reagent Program. An HIV-positive patient serum was used for the identification of HIV-1 capsid (CA) protein.

### Immunoblotting

For immunoblot analysis of intracellular proteins, whole cell lysates were prepared as follows: Cells were washed once with PBS, suspended in PBS and mixed with an equal volume of sample buffer (4% sodium dodecyl sulfate, 125 mM Tris-HCl, pH 6.8, 10% 2-mercaptoethanol, 10% glycerol, and 0.002% bromphenol blue). To analyze virus-associated proteins, cell-free filtered supernatants from transfected HeLa cells (5–6 ml) were pelleted (75 min, 35,000 rpm) through a 20% sucrose cushion (4 ml) in an SW41 rotor. The concentrated virus pellet was suspended in PBS and mixed with an equal volume of sample buffer. Proteins were solubilized by heating 10 to 15 min at 95°C. Cell and virus lysates were subjected to SDSPAGE; proteins were transferred to PVDF membranes and reacted with appropriate antibodies as described in the text. Membranes were then incubated with horseradish peroxidase-conjugated secondary antibodies (Amersham Biosciences, Piscataway NJ) and proteins were visualized by enhanced chemiluminescence (ECL, Amersham Biosciences).

### Virus preparation

Virus stocks were prepared by transfection of HeLa cells with appropriate plasmid DNAs. Virus-containing supernatants were harvested 24 h after transfection. Cellular debris was removed by centrifugation (3 min, 1500 rpm) and clarified supernatants were filtered (0.45 μM) to remove residual cellular contaminations. Filtered virus stocks were further purified and concentrated by pelleting through 20% sucrose (75 min, 4°C at 35,000 rpm in an SW41 rotor).

### Viral infectivity assay

To determine viral infectivity, virus stocks were normalized for equal reverse transcriptase activity and used to infect 5 × 10^5 ^LuSIV cells [51] in a 24-well plate in a total volume of 1.2 to 1.4 ml. Infection was allowed for 24 h at 37°C. Cells were then harvested and lysed in 150 μl of Promega 1x reporter lysis buffer (Promega Corp., Madison WI). To determine the luciferase activity in the lysates, 50 μl of each lysate were combined with luciferase substrate (Promega Corp., Madison WI) by automatic injection and light emission was measured for 10 seconds at room temperature in a luminometer (Optocomp II, MGM Instruments, Hamden CT).

### Immunofluorescence and confocal microscopy

HeLa cells were transfected as indicated in the text. Transfected cells were trypsinized and single-cell suspensions were distributed into 12 well plates containing 0.13 mm cover slips. Cells were grown for 15 h at 37°C in DMEM containing 10% FBS. Cells were fixed at -20°C in precooled methanol (-20°C) for 10 minutes followed by two washes in PBS. For antibody staining, coverslips were incubated in a humid chamber at 37°C for 1 hr with primary antibodies at appropriate dilutions in 1% BSA in PBS. Coverslips were washed once in PBS (5 min, room temp) and incubated with Cy2-conjugated secondary antibodies (diluted in 1% BSA in PBS) for 30 min at 37°C in a humid chamber. Coverslips were then washed twice with PBS and mounted onto microscope slides with glycerol gelatin (Sigma-Aldrich Inc., St. Louis MO) containing 0.1 M N-propyl gallate (Sigma) to prevent photo bleaching. For confocal microscopy, a Zeiss LSM410 inverted laser scanning microscope equipped with a krypton/argon mixed-gas laser was employed. Images were acquired with a Plan-Apochromat 63x/1.4 oil immersion objective (Zeiss). Image quality was enhanced during data acquisition using the LSM line average feature (8x). Post-acquisition digital image enhancement was performed using the LSM software.

### Sucrose step gradient analysis

Sucrose step gradients were prepared as follows: 2.0 ml of a 60% sucrose solution (in PBS) was placed into the bottom of SW55 centrifuge tubes and overlaid with 2.1 ml of a 20% sucrose solution. Immediately prior to addition of concentrated virus stocks (500 μl), the step gradients were overlaid with 100 μl of either PBS or 1% Triton X-100. This procedure minimized the time of detergent exposure of the virus. Samples were then centrifuged in a SW55Ti rotor (Beckman) for 60 min at 35,000 rpm and 4°C. Three fractions (S1, S2, S3) of 1.1 ml each were collected from the top. Aliquots of each fraction of step gradients were subsequently processed for immunoblotting.

## Competing interests

The author(s) declare that they have no competing interests.

## Authors' contributions

RG conceived the study, was leading the execution of the experiments, and participated in the writing of the manuscript. KS coordinated and supervised the study and was involved in the writing of the manuscript. MK, EM, and SK participated in virus production and sample preparation and provided critical comments on the manuscript.
